# Giving offspring a healthy start: parents' experiences of health promotion and lifestyle change during pregnancy and early parenthood

**DOI:** 10.1186/1471-2458-11-936

**Published:** 2011-12-15

**Authors:** Kristina Edvardsson, Anneli Ivarsson, Eva Eurenius, Rickard Garvare, Monica E Nyström, Rhonda Small, Ingrid Mogren

**Affiliations:** 1 Department of Public Health and Clinical Medicine, Epidemiology and Global Health, Umeå University, SE 901 87 Umeå, Sweden; 2 Mother and Child Health Research, La Trobe University, Melbourne, Victoria 3000, Australia; 3 Division of Quality Management, Luleå University of Technology, SE 971 87 Luleå, Sweden; 4 Medical Management Centre, Department of Learning, Informatics, Management and Ethics, Karolinska Institutet, SE 171 77 Stockholm, Sweden; 5 Department of Psychology, Umeå University, SE 901 87 Umeå, Sweden; 6 Department of Clinical Science, Obstetrics and Gynecology, Umeå University, SE 901 87 Umeå, Sweden

## Abstract

**Background:**

There are good opportunities in Sweden for health promotion targeting expectant parents and parents of young children, as almost all are reached by antenatal and child health care. In 2005, a multisectoral child health promotion programme (the Salut Programme) was launched to further strengthen such efforts.

**Methods:**

Between June and December 2010 twenty-four in-depth interviews were conducted separately with first-time mothers and fathers when their child had reached 18 months of age. The aim was to explore their experiences of health promotion and lifestyle change during pregnancy and early parenthood. Qualitative manifest and latent content analysis was applied.

**Results:**

Parents reported undertaking lifestyle changes to secure the health of the fetus during pregnancy, and in early parenthood to create a health-promoting environment for the child. Both women and men portrayed themselves as highly receptive to health messages regarding the effect of their lifestyle on fetal health, and they frequently mentioned risks related to tobacco and alcohol, as well as toxins and infectious agents in specific foods. However, health promotion strategies in pregnancy and early parenthood did not seem to influence parents to make lifestyle change primarily to promote their own health; a healthy lifestyle was simply perceived as 'common knowledge'. Although trust in health care was generally high, both women and men described some resistance to what they saw as preaching, or very directive counselling about healthy living and the lack of a holistic approach from health care providers. They also reported insufficient engagement with fathers in antenatal care and child health care.

**Conclusion:**

Perceptions about risks to the offspring's health appear to be the primary driving force for lifestyle change during pregnancy and early parenthood. However, as parents' motivation to prioritise their own health per se seems to be low during this period, future health promoting programmes need to take this into account. A more gender equal provision of health promotion to parents might increase men's involvement in lifestyle change. Furthermore, parents' ranking of major lifestyle risks to the fetus may not sufficiently reflect those that constitute greatest public health concern, an area for further study.

## Background

The opportunity for health promotion targeting expectant parents and parents of young children are seemingly good in Sweden, since almost all, regardless of social group or ethnicity, are reached by health services during pregnancy and early parenthood [1,2]. In general, expectant parents and parents of young children are satisfied with the Swedish antenatal care (ANC) [3,4] and child health care (CHC) services [5,6], and the attendance rates, close to 100%, are high from an international perspective [1,2].

Currently, half of Swedish women and two thirds of men report not meeting national recommendations on healthy lifestyle [7]. On the other hand, many want to improve their way of life and also want to be supported to make lifestyle changes [7]. Particularly important target groups for health promotion efforts are those expecting a baby and entering parenthood, as their health and lifestyle will influence their offspring's health and development throughout the life course [8-10] and they have significant contact with health and children's services at this life stage. Furthermore, the family environment, including parenting styles and attitudes towards eating habits and physical activity, lay the foundation for children's health-related behaviours [11-14]. Fathers have become increasingly important in this context because of the trend to increased paternal responsibility and involvement in child care. Today, child care duties are shared more equally between Swedish mothers and fathers than other household duties, reaching an almost equal share when women return to paid work full time [15]. Shared parental responsibility as well as gender equity are encouraged by the Swedish Government [16]. A bonus in the parental insurance system was launched in 2008 to support a more gender equal sharing of parental leave and a subsequent more equal participation in working life [16]. In 2010, the 480 days of parental leave in Sweden were shared 77% by women and 23% by men [17]. Internationally, Sweden is regarded among the countries most supportive of fathers' involvement in child care. Other examples of countries that have extended paternity leave with high income replacement are Canada, Finland, Germany, Iceland, Norway, Portugal, Slovenia and Spain [18].

The organization of ANC and CHC in Sweden entails opportunities for regular contacts with expectant parents and parents of young children. Promotion of a healthy lifestyle is an important part of this interaction [19,20]. Although considerable evidence of health benefits exists, supporting people to adopt healthier lifestyles is challenging [7]. Theories and models for understanding how change occurs have been developed [21], but effective healthy lifestyle interventions at the population level remain to be found. In parallel to individual level factors, social and structural factors are important to consider as these have a major influence on health and health-related behaviour [22]. This is illustrated by the fact that poor health and unhealthy lifestyles follow a stepwise social gradient, with greater disadvantage evident further down the social ladder [7,22].

Evaluation of health promotion is complex, especially when interventions provided are expected to achieve measurable improvements several years or sometimes even decades after their initiation. However, intermediate measures are important and can be fruitfully explored using qualitative methods [23]. Allowing for investigation of the process of change and what is happening in the 'black box' between input and outcome [23], qualitative research methods can also assist in examining the reasons people do or do not adopt healthy lifestyle behaviours [24]. Several studies have previously addressed various aspects of women's and men's experiences during pregnancy and the transition into parenthood [25-30], but none to the best of our knowledge, has specifically given attention to parental experiences of health promotion and lifestyle change during this time period. Thus, the aim of the present study was to explore Swedish first-time parents' experiences of health promotion and lifestyle change during pregnancy and early parenthood.

## Methods

### The setting

#### Description of the core sectors involved

Swedish pregnant women are normally enrolled in ANC in the first trimester of pregnancy where midwives are responsible for maternal and fetal surveillance including counselling [19]. The basic antenatal programme includes seven to nine visits during pregnancy with a follow up visit postpartum [19]. If complications occur during pregnancy, the woman is referred by the midwife to a family physician or an obstetrician for counselling [19]. A child health nurse generally visits the family in their home within a week of birth. The primary aim of this visit is to establish good contact with the family after which they are invited to attend the CHC centre for health and development check-ups, immunizations, support, information, and advice. Approximately 11 visits are scheduled during the child's first 18 months, with further visits at 3, 4, and 5 years of age including some involving an examination of the child by a physician [20]. Childbirth and parenthood classes are arranged within ANC and CHC. Open pre-schools serve as alternatives to regular pre-schools for children with parents on parental leave or not in the paid workforce. Parents and children aged 0-5 years attend together on a drop-in basis. There is no registration and participation is entirely voluntary [31]. Visits to ANC, CHC and pre-schools in Sweden are all free of charge, and dental care is free for children up to the age of 19 years [32].

#### The Salut programme--a multisectoral child health promotion programme in Västerbotten

In 2005, Västerbotten County Council in northern Sweden launched the Salut Programme in an effort to develop and strengthen health-promoting activities in ANC, CHC, dental services, social services, and school settings [33,34]. Starting with the pregnant woman and her partner, the programme follows the child up to 18 years of age via age-specific interventions. The interventions are integrated with ordinary work tasks and summarized in manuals, protocols, and questionnaires specific to each sector. In ANC and CHC, interventions include suggesting strategies such as physical activity, a healthy diet, discussing body mass index (BMI) and weight gain (during pregnancy), tobacco, alcohol, drugs, dental health (free dental care visit offered via ANC), domestic violence (screening in ANC and CHC), psychological well-being, social support, parenting, and parent relationships. The interventions targeting parents and their children from fetal life to 18 months of age are described in more detail elsewhere [33].

### Study design

The study was carried out in four pilot areas where the Salut Programme was first established within the regular ANC, CHC, dental services and open pre-schools. These areas were selected to be representative of the overall demographic structure of the county. A qualitative study design was adopted using in-depth semi-structured interviews with first-time mothers and fathers when their child had reached 18 months of age. Qualitative manifest and latent content analysis was undertaken, which meant analyses of what the text said (manifest content) and an interpretation of the underlying meaning of the text (latent content) [35].

### Informants

Twenty-four informants (12 couples) were recruited, while nine eligible informants declined participation. The inclusion criterion was being a first-time mother or father (independent of relationship status) of an 18 month-old child. All informants were cohabiting or married, and of Swedish origin, except one female who had immigrated from Southeast Asia 2 years previously. The sample included informants with a range of lifestyles and weights (no informant was perceived obese or underweight by the interviewer), including women and men using and not using snuff, cigarettes and alcohol, and ranging from not very to very physically active. Nearly half the mothers were currently pregnant with their second child at the time of the interview. Socio-demographic characteristics of informants, as well as their reported interactions with the involved sectors, are shown in Tables 1 and 2.

**Table 1:** Socio-demographic characteristics of informants (*n *= 24) in Västerbotten, Sweden

**Couple no**.	Females		Males		Living area
	**Age (yrs)**	**Highest education**	**Age (yrs)**	**Highest education**	

1	28	Secondary school^1^	27	Secondary school	Rural area
2	31	Secondary school	26	Secondary school	Rural area
3	25	Secondary school	29	Secondary school	Village
4	33	Secondary school	30	Secondary school	Village
5	31	University^2^	30	University	Small town
6	31	Secondary school	34	University	Small town
7	33	University	31	University	Small town
8	29	University	35	University	Small town
9	35	University	34	University	Suburb
10	32	Secondary school	31	Secondary school	Suburb
11	35	University	39	University	Suburb
12	32	Secondary school	46	Secondary school	Suburb

Occupations are listed in alphabetical order to secure informant confidentiality.

**Female: **Administrator, banking adviser, granted sick leave, hair dresser, industrial worker, manual worker, nurse (*n *= 2), office employee, pre-school teacher, primary school teacher and university project assistant.

**Male: **Accountant consultant, computer engineer, consultant worker, finance manager, high school teacher, industrial purchaser, industrial worker (*n *= 2), manager, programmer and salesman.

^1^12 years of schooling

^2^15-17 years of schooling

**Table 2:** Informants' encounters with involved sectors during pregnancy and the child's first 18 months

**Couple no**.	Females					Males				
	
	Visits to ANC^1^	Visits to CHC^2^	Participation in childbirth and parenthood classes	Visit to dental hygienist during pregnancy	Ever visited open pre-school	Visits to ANC	Visits to CHC	Participation in childbirth and parenthood classes	Visit to dental hygienist during pregnancy	Ever visited open pre-school
1	Most visits	Most visits	ANC and CHC	No	Yes	Most visits	Sometimes	Only ANC	No	Yes
2	Most visits	Most visits	Only ANC	No	Yes	Most visits	Sometimes	Only ANC	No	Yes
3	Most visits	Most visits	Only CHC	No	Yes	Most visits	Most visits	No	No	Yes
4	Most visits	Most visits	ANC and CHC	Yes	Yes	Most visits	Most visits	Only ANC	Yes	No
5	Most visits	Most visits	Only ANC	Yes	Yes	Most visits	Most visits	Only ANC	Yes	Yes
6	Most visits	Most visits	Only CHC	No	Yes	Most visits	Most visits	Only CHC	No	Yes
7	Most visits	Most visits	Only ANC	No	Yes	Sometimes	Seldom	Only ANC	No	No
8	Most visits	Most visits	Only ANC	No	Yes	Sometimes	Sometimes	Only ANC	No	Yes
9	Most visits	Most visits	ANC and CHC	No	Yes	Most visits	Most visits	ANC and CHC	No	Yes
10	Most visits	Most visits	Only ANC	No	No	Most visits	Most visits	Only ANC	No	No
11	Most visits	Most visits	ANC and CHC	No	Yes	Sometimes	Sometimes	ANC and CHC	No	Yes
12	Most visits	Most visits	Only ANC	Yes	Yes	Seldom	Seldom	ANC (once)	No	Yes

^1^Antenatal care

^2^Child health care

### Data collection procedures

Eight child health care nurses working at four CHC centres were asked to invite all parents meeting the inclusion criteria for participation in the study at the child's routine 18-month visit. Parents who agreed on participation were given written information about the study by the nurse, and were thereafter contacted by the first author (KE) via telephone for further information and agreement of a suitable time for the interview. Informed consent was established verbally during the phone call. Informants were recruited from June to December 2010. The interviews were conducted by the first author in the homes of the informants within a couple of weeks of recruitment. The mother of Southeast Asian origin was interviewed in English on request. A semi-structured interview guide developed by the research team was piloted during the first two interviews and thereafter slightly revised (Table 3). All interviews were carried out separately with women and men and were digitally recorded. They lasted between 23 and 58 min (mean time 39 min). Demographic characteristics were collected prior to each interview and general impressions and reflections were noted directly afterwards. A total of 30 study informants were aimed for a priori. After preliminary analyses of the first 20 interviews, data saturation was assessed. Even though collected data were perceived as saturated in relation to the research question, a further four individual interviews were undertaken to ensure the inclusion of a broad variety of experiences. However, as no more substantial ideas or concepts emerged during the last interviews, further recruitment of informants was stopped.

**Table 3:** Key domains in the interview guide

Key domains^1^

• Experiences from ANC^2^, CHC^3^, dental services, and open pre-schools (including childbirth and parenthood classes within ANC and CHC)
• The involvement of mothers and fathers in each sector
• Experiences of health promotion initiatives in each sector
• Perceptions of own and families' health and lifestyles
• Changes of lifestyle from start of pregnancy to current date
• Reasons behind and barriers for lifestyle change
• Sources of health information
• Experiences of the Salut Programme including involvement in programme specific interventions

^1^All questions covered the time from start of pregnancy to the time for the interview. Additional probing questions were used to encourage informants to elaborate their responses and narratives.

^2^Antenatal care

^3^Child health care

### Data analysis

Interviews were transcribed verbatim and analysed with qualitative manifest and latent content analysis [35]. First, all interviews were read several times by the first and last author (KE and IM) to get a sense of the whole. Both authors noted key emerging ideas and concepts during this process. Second, all interviews were coded by the first author using the software Open Code 3.6 [36] and selected interviews were also coded by the last author. The coding was then compared and was found to be largely consistent. Third, all codes were compared, and similar codes were grouped in content areas and then abstracted into sub-categories and categories. Codes from men and women were separately coloured in this process so that their experiences could be adequately accounted for. However, separate coding schemes were not found necessary as the same pattern of topics occurred independent of gender. Thus, the categories were based on codes representing both women and men. The whole analysis process entailed a back and forth movement between the interviews, codes, sub-categories, categories, and themes. It also involved close collaboration between the first and the last author, and uncertainties in coding and interpretation were thoroughly discussed until consensus was obtained. Fourth, the preliminary results were reflected upon and discussed by the research team and then slightly revised. A set of 16 sub-categories and their six categories was finally agreed upon, with two main themes unifying these. Finally, a researcher (RS) not involved in data collection and analysis reviewed data, the process of analysis and the interpretations made, which resulted in minor revisions.

### Ethical considerations

All participation was voluntary and informed consent was obtained verbally from all participants prior to the interviews. Informant confidentiality was thoroughly discussed during the research process and highly prioritised in reporting of findings. Highly identifiable characteristics of informants are not included in text and tables, and the reference 'mother' or 'father' is used in presentation of quotations. The study was carried out in compliance with the ethical principles presented in the Helsinki Declaration. Ethical approval was obtained from the Regional Ethical Review Board in Umeå, Sweden, Dnr 2010-63-31.

## Results

Overall, the informants expressed confidence in the Swedish Health Care System, as providing them high access, good support and offering a 'safety net' during pregnancy and early parenthood. Professionals in involved sectors were generally perceived as competent, experienced, knowledgeable and a good source of information and support when uncertainties or problems were encountered. The Salut Programme was recognized by almost all mothers and they associated the programme with diverse health promotion activities. However, most fathers did not recognize the programme at all, and few had participated in the specific component directed only to them, a father's visit in CHC.

Two themes emerged during analysis: I) 'Experiencing healthy lifestyle promotion without own lifestyle change', with three categories and nine sub-categories, and II) 'Offspring's health as a primary incentive for lifestyle change', with three categories and seven sub-categories (Table 4). The reported findings refer in large part to ANC and CHC, as the informants perceived that their experiences of health promotion activities from the other sectors were limited. Below, the themes are presented in bold font, categories in bold italics and sub-categories in italics. The quotations provided throughout the text represent a variety of informants.

**Table 4:** Themes, categories and subcategories describing parents' experiences of health promotion and lifestyle change during pregnancy and early parenthood

Themes	Categories	Sub-categories
I. Experiencing healthy lifestyle promotion without own lifestyle change	Encountering superficial health promotion	Healthy lifestyle is common knowledge
		Experiencing lack of parental attention when entering child health care
		Experiencing health promotion advice as too directive
		Appearing healthy means no questions and advice
	Facing prevailing traditional gender roles	As partner being the third person of interest
		Perceiving ANC^1 ^and CHC^2 ^as 'women's business'
	Feeling no urgency to prioritise own health	Feeling strong and healthy
		Ranking risks and postponing lifestyle change
		Relapse after pregnancy and breastfeeding
II. Offspring's health as a primary incentive for lifestyle change	Securing the health of the fetus	Avoiding fetal risk exposure
		Adhering to guidelines on healthy living
		Taking shared or single responsibility
	Providing a health promoting environment for the child	Being a role model
		Changing daily routines and own lifestyle
	Setting priorities for own health	Maintaining a healthy lifestyle to avoid adverse outcomes
		Facing barriers to healthy living

^1^Antenatal care

^2^Child health care

### Theme I--experiencing healthy lifestyle promotion without own lifestyle change

#### Encountering superficial health promotion

##### Healthy lifestyle is common knowledge

Promotion of a healthy lifestyle because of the pregnancy was experienced as a taken for granted part of ANC, and at CHC visits, in relation to child health. However, the informants did not report receiving new messages on parental health and lifestyle from the involved sectors. As the basic concept of 'healthy living' was perceived as common knowledge, what was communicated did not strike the informants as anything new.

Well, I felt, it was the same [messages we got] in home economics classes in high school, it was like the same things. So, you know how to have a healthy lifestyle, but ... ah, it just doesn't happen... (Father)

##### Experiencing lack of parental attention when entering child health care

The focus and promotion of a healthy lifestyle during pregnancy was described as being partly lost in the transition from ANC to CHC. In CHC the focus was perceived as almost solely directed towards the child, and parents felt their own health issues were not attended to. Some parents found visits to CHC to be standardized and time constrained, something that resulted in their avoidance of raising issues and also to feelings of insufficient engagement from health professionals.

Certainly, everyone has their ups and downs psychologically. But, what I would like CHC to do more of is to pay attention to, and care more about mothers. There is so much focus on the children, their development. But, just asking the mothers, how are you? Hardly anybody does that. (Mother)

I sometimes get the feeling that CHC is like a conveyor belt, they are so terribly busy, so, things are just ticked off, the things that are to be done, so it's all correct in their system. (Mother)

##### Experiencing health promotion advice as too directive

Although many informants reported positive aspects of their encounters in ANC and CHC, negative experiences were also evident. Advice was sometimes experienced as too directive and preachy, and too standardized. This led participants to experience health messages as redundant, annoying, guilt provoking or making them feel like hiding their concerns.

In my opinion it depends on how things are presented. Because if it is presented as a sermon, then you probably will get annoyed that people want to control how you raise your children and the way you live your life. (Mother)

I even think she said [the midwife] 'you should not' or 'you must not', but then of course you do whatever you want with this information anyway. But the best for your child and your conscience is to follow it'. (Mother)

##### Appearing healthy means no questions or advice

Some informants believed that they were not asked questions about their lifestyle as they presented as being healthy. Further, by indicating good health and a healthy lifestyle when filling in questionnaires, both female and male informants reported that they received little subsequent counselling about health and lifestyle.

But I'm quite convinced, if I was extremely overweight or did look very sick, she probably would have asked [about my lifestyle]. (Mother)

#### Facing prevailing traditional gender roles

##### As partner being the third person of interest

A fairly common experience among fathers but also reported by some mothers, was that men were being treated as fairly unimportant in the ANC and CHC settings. This was based on the informants' interpretations of the professionals' verbal approaches, or body language. Not feeling important or welcomed resulted in some men feeling less involved. When midwives and child health nurses directed questions and their conversation to both parents, the men's sense of participation increased.

My experience is that the professionals in CHC only turn to my partner when they are talking; they only look at her, and not so much at me. That makes me feel a little bit less involved. (Father)

Very few men reported that their lifestyle had attracted attention in encounters in ANC and CHC. Furthermore, the way parenthood classes were organized was also perceived as excluding men, particularly in CHC, since meetings arranged during the day posed a major barrier to their participation. Motivation was further lowered as very few male peers showed up at these meetings. While some fathers perceived this allocation of responsibilities between women and men as natural, others perceived 'traditional' attitudes as provocative.

Parenthood classes were always arranged in the evenings before the children were born, and this enabled everyone to participate. Both mothers and fathers. Then when CHC took over, the classes were arranged during the day, which resulted in only mothers attending. (Mother)

I think it mirrors the way of thinking in antenatal care, that men are, we're not really part of the game.... There is a need, it would be better if, because I mean, as a man, you are equally important in that context, really. (Father)

##### Perceiving antenatal care and child health care as 'women's business'

Most men described an interest in following their child's development and taking responsibility already during pregnancy. However, some men perceived ANC and CHC to be 'women's business' and stated no major interest in becoming involved.

I think it seems that it's mostly for the ladies. My partner seemed to find it quite enjoyable to go there, but I had no interest in going there at all.... [to the CHC centre] (Father)

#### Feeling no urgency to prioritise own health

##### Feeling strong and healthy

In general, the informants perceived themselves as being healthy and having a healthy lifestyle, even though some said that they probably would benefit from weight loss, more physical exercise, a healthier diet, or from quitting using snuff. However, motivation for lifestyle change was low among informants because they felt healthy.

Well, it is certainly difficult to find motivation. It would be different if a doctor told me that 'You've got a problem with your cholesterol' but, as long as I feel good.... (Father)

##### Ranking risks and postponing lifestyle change

The potential for health problems, due to previous or current unhealthy lifestyle, did not commonly seem to raise concern among the informants. Health problems, caused by over consumption of energy dense foods, and soft drinks or lack of exercise, were perceived by some as reversible, and therefore something that could be tackled later. However, a ranking of risks concerning their own health was evident in some narratives.

Reduced life expectancy would make me change quite drastically; and physical decline would also make me change. And, the fact that 'smoking kills' as well, there is no way in the world I would start smoking. It's unthinkable. (Father)

I don't know how to express myself, but I don't feel urgently in need of changing anything... [laughs]. (Father)

##### Relapse after pregnancy and breastfeeding

Relapsing to pre-pregnancy lifestyle after birth and breastfeeding was commonly reported among the mothers. Examples were use of snuff, consumption of sugar products, or weight-gain when being less strict with dietary habits. Incentives for continuing a healthy lifestyle became weaker when the perceived health risks exposing the fetus were no longer present. A few men, who had changed habits to support their partners during pregnancy, described relapsing to their old lifestyle simultaneously with their female partner. This pattern revealed a high awareness among the informants of the influence of lifestyle on fetal health.

I kept away from snuff as long as I was breastfeeding. But when I stopped breastfeeding, it didn't take long before I was hooked again. (Mother)

### Theme II--offspring's health as a primary incentive for lifestyle change

#### Securing the health of the fetus

##### Avoiding fetal risk exposure

Women described the ease with which they made lifestyle changes as strongly linked to their perceptions about health risks to the fetus. The higher perceived risk to the fetus, the easier the change. When confirmed pregnant, and prior to first contact with antenatal care, most women instantly made lifestyle changes to protect the health of the fetus. A positive pregnancy test was described as a significant event promoting a decision to quit using snuff, cigarettes and alcohol.

I had no other choice than to stop [smoking and using snuff], that's how I see it. Because everything goes straight into the baby. (Mother)

##### Adhering to guidelines on healthy living

Female informants, but also some men, described themselves as highly receptive to information regarding how fetal health could be affected by the lifestyle of the woman. Fetal health risks associated with use of tobacco and alcohol were seen as general knowledge and natural to avoid. Both female and male informants clearly recalled dietary advice from ANC. They frequently recited specific recommendations, mostly risks regarding toxins and infectious agents associated with consumption of specific foods. In general the dietary advice was reported as easy to adhere to. There were some though, who experienced it as too detailed and even exaggerated, especially when expressed in terms of what pregnant women were allowed to eat or not.

There are some things you shouldn't eat, as it can harm the fetus and the child, and inhibit development. And, I haven't t been drinking wine or anything like that, as that's not good either. Well, I have also been thinking about sweets, you shouldn't eat too many sweets either during pregnancy and breastfeeding, because it will also be transferred to the child. (Mother)

My partner wasn't allowed to eat just anything during pregnancy. She was told to be careful with freshwater fish and things like that. To keep a balanced diet, and to avoid alcohol and smoking. (Father)

Some informants found that judgements about nutritional intake related to fetal risks were difficult to make and for that reason they appreciated explicit advice. However, some women said that they wouldn't adhere to dietary advice as strictly during subsequent pregnancies. By developing confidence and knowledge, they felt that in future they would more easily assess lifestyle risks for the fetus.

The first time I was pregnant, I found it hard to know what was at stake, how dangerous things really were. What are the chances that I might harm the baby by eating a certain thing? You have no idea the first time. But, I have a better idea now. (Mother)

While a few women mentioned receiving recommendations on weight gain in pregnancy, they generally did not reflect on the associated maternal and fetal risks. The topic's inherent sensitivity was touched upon by examples of friends becoming upset when being approached about their weight. One mother suggested that an objective discussion of fetal risks associated with maternal overweight would be a better starting point than'blaming' the mother for weight gain.

A friend of mine who is extremely overweight got taunts [from the midwife]... and she experienced a kind of undertone... That makes me sick really. Even if she's overweight, she shouldn't be met by taunts or sneers from professionals. Instead, they should have had a serious and helpful conversation [about it]. (Mother)

##### Taking shared or individual responsibility

Some women and men saw the pregnancy as a shared responsibility as they perceived men's roles to be equally important. By accompanying the pregnant woman to antenatal care, men were also recipients of health messages and advice on lifestyle. A few men described changing their own habits in order to support their partner's dietary changes during pregnancy.

Well, it's his child too. It's me who was pregnant but we were pregnant. That's how we looked at it, that WE were pregnant. (Mother)

I think it's mostly for the women [involvement in ANC and CHC]. I really do. But nowadays everything is supposed to be as equal as possible. An [uneven] gender distribution is not allowed in our Swedish society. But there's not much you can do about that; you just have to accept it. (Father)

#### Providing a health promoting environment for the child

##### Being a role model

Becoming a parent made the informants reflect on their lifestyle and in what way their own habits could influence the health of their child. Many perceived their own lifestyle to have been formed in childhood. Setting a good example therefore became important, as their own behaviour was perceived as strongly linked to the behaviour their children would adopt. The importance of being a role model in shaping a healthy environment for the child was frequently mentioned.

I have to start eating more vegetables and stuff like that to show that [laughs] this is something that you actually can eat. I was brought up with a dad who never ate any vegetables, so neither did I or my brother. (Father)

##### Changing daily routines and own lifestyle

As children left infancy and became toddlers, parents described lifestyle changes as regaining importance. In particular, changes related to dietary habits, like including vegetables in meals, having regular family mealtimes, and reducing intake of snacks, sweets and soft drinks. Using snuff or spending lot of time in front of TV or on the computer was viewed as having a negative influence on children. Even though some informants described benefits for themselves based on the changes made, change was reported mostly as for the child's good and not their own.

When it's only about yourself, then, I'm less concerned. But, our child has encouraged us to be more careful because we want her to have a healthy diet. And if she is to have a healthy diet, she must see that we do too. So that has helped us to become healthier as well. (Mother)

The only habit I think I've changed is that I try not to sit in front of the computer when the child is present. So that he won't do the same thing. (Father)

#### Setting priorities for own health

##### Maintaining a healthy lifestyle to avoid adverse outcomes

Some informants described how their experiences of adverse outcomes of 'unhealthy choices' made them strive to maintain a healthy lifestyle to promote their own health. Some revealed that they kept up regular physical activity to avoid problems such as fatigue, depressed mood, or overweight.

If I don't exercise, I get very tired. Then I just get sick of everything. So I have to try to keep up my fitness in order to sustain my mental wellbeing. (Father)

##### Facing barriers to healthy living

Time constraints or tiredness were commonly experienced as barriers for healthy choices, especially when parental leave was over. Preparing healthy, home cooked meals or doing exercise became less of a priority. Living in rural areas and having to commute long distances to reach sports facilities restricted exercise opportunities for some. Furthermore, the climate in the north of Sweden was also mentioned as limiting opportunities for being physically active during winter.

The hardest thing has been to get some regular exercise when you're at home with a child, or now when you're working and being tired after a long day. Then you have to go home and cook and play with your child until bedtime, and then you are completely exhausted. (Mother)

## Discussion

Our main findings were that parents made lifestyle changes to secure the health of the fetus during pregnancy, and in early parenthood, to create a health-promoting environment for the child. These findings suggest that pregnancy and early parenthood represent important opportunities for promoting healthy lifestyle change. However, health promotion messages less often influenced the informants to make lifestyle changes with the aim of promoting their own health. Several alternative explanations for this include already having a healthy lifestyle, not being reached by the interventions as intended, or that the interventions were not sufficiently tailored to meet the needs of parents at this time.

We found that parents were highly receptive to health messages regarding the effect of their lifestyle on fetal health. To a large extent couples reported the risks associated with tobacco, alcohol, and risks related to toxins and infectious agents in specific foods. Both mothers and fathers took action to avoid such risks in order to secure the health of the fetus, with the highest perceived risk provoking the easiest change. It was surprising however, that risks associated with overweight and obesity were scarcely mentioned, even though 37% of Swedish women are in fact overweight or obese at the start of pregnancy, something which entails risks of several adverse pregnancy outcomes for the mother [37,38], and the infant [37-40]. These findings are even more surprising considering that a maternal BMI of 25 or more is currently estimated to be the most important modifiable risk factor for stillbirth in high income countries [40]. One reason for the lack of discussion of risks associated with overweight and obesity could possibly be related to the composition of our participant group, none of whom were regarded as being obese at the time of the interview. However, the awareness of other risks as mentioned was seemingly high, even among those where it was evidently not part of their lifestyle. Thus, there are reasons to broaden the range of risk factors to also include those related to contemporary lifestyles, such as maternal overweight. A normal weight development and a return to pre-pregnancy weight after birth (thus also preventing overweight during subsequent pregnancies) have long been included in the goals of ANC in Västerbotten, and also a prioritised goal within the Salut Programme with its emphasis on healthy lifestyle interventions. However, it may be that the sensitivity of the topic still hinders health care providers from adequately addressing the issue [41-43], and a question is whether parents would be as motivated to take steps to modify these well-established risks if they were just as well communicated as those to do with avoidance of certain foods. This is a very important area for further research. In addition, for the benefit of the woman and her future children, the ideal would be to promote a healthy lifestyle and address overweight before conception. However, the area of routine pre-pregnancy health promotion is sparsely studied and evidence for the effectiveness of such interventions is still lacking [44].

This study adds to the literature about the challenges for health promoting interventions directed to low-risk patients or general populations [45,46], considering the findings that parents did not seem to initiate changes with the primary aim of promoting their own health. These findings are consistent with theories developed to assist in the understanding of behavioural change. The Health Belief Model suggests that the threat of disease or negative outcome is a key determinant when individuals make health behaviour change, weighing up the seriousness of disease, perceived barriers to and benefits of change [47]. Thus, people who are unconcerned about their health or other associated negative outcomes are unlikely to adopt new behaviours [47]. This is something that has to be considered in the design of health promotion programmes targeting expectant parents and parents of young children, when improvements are sought in both child and parent outcomes. Moreover, even if the health of the offspring seems to be a driving force for healthy lifestyle change, it is important to bear in mind that a healthy lifestyle is created within the family. Consequently, in promoting child health, the whole family environment needs to be supported.

The parents in our study described their own needs, health and lifestyles as being somewhat neglected in child health care, as the primary focus was on their child. This is consistent with previous findings that Swedish mothers are satisfied with the CHC's services directed to the child, while they perceive that their own needs are not sufficiently recognized [5]. The high attendance rate in parenthood classes in ANC and low attendance rate in CHC among the male informants is in accordance with what previously has been reported in Sweden [48]. Several previous studies have also described fathers' experiences of not having their needs met in the female-oriented antenatal, postnatal and child health care settings, even when they seek to be involved [26,28,30,49-52]. Our results are consistent with these previous findings, and instead of further studies in this area, it is time to translate this knowledge into action. In health promotion, it is plausible that positive outcomes cannot be expected on gender-equal terms if fathers are not reached by interventions, given that effective health promotion strategies are at hand. This is particularly important to consider in the light of the fact that risk factors for poor health, such as overweight and obesity, low intake of fruit and vegetables, sedentary lifestyle, overuse of alcohol and use of snuff are more common among men [7,34].

A 'one size fits all' approach in health promotion entails overlooking individual needs or incentives for change even though this strategy can be useful when the target population shares the same information needs [53]. Health messages tailored for a particular individual, such as computerized algorithms, computerized nutritional education programmes or the use of risk assessment and family history information have been shown to be more effective than generic healthy lifestyle promotion messages [54-56]. However, lifestyle counselling is a balancing act. Directive, confrontational approaches and unsolicited advice can lead to resistance among recipients [57], as our study found. Client-centred counselling styles have been shown to be favourable when adherence to health care advice is sought [57]. Motivational interviewing (MI) is one successful client centred counselling approach [57,58], nowadays applied in various fields of health behaviour change [58], and also introduced via short courses in ANC and CHC in Västerbotten. The likelihood of positive outcomes of MI-based counselling rises with increasing number of encounters as well as the encounter's duration [58]. Thus, the antenatal and child health care setting constitutes a platform for enhanced health promotion due to regular contact with parents for five to six years after enrolment, and even longer when there are subsequent pregnancies.

Even though health promotion is an integral part of the Swedish health care system, the Västerbotten County Council aimed to strengthen this effort with the initiation of the Salut Programme to combat public health problems such as the rising obesity epidemic and worrying trends in dental health. However, to understand the outcomes of such an intervention, the 'black box' between input and outcome has to be explored. One approach is to examine facilitators and barriers to adherence at different levels of health care [59]. This study illuminates factors related to the recipients of interventions: the parents. We have in a previous study shed light on factors experienced by the individual professionals in which several barriers to adherence to the programme were found. Among these, time constraints were frequently reported, particularly by professionals in child health care [33]. This might be one explanation for the insufficient involvement of fathers, the lack of a holistic approach in health promotion strategies and the directive counselling styles, as professionals lack sufficient time to engage in more client-centred dialogues. However, more research is needed to get a comprehensive understanding of how success in health promoting efforts can be achieved.

### Trustworthiness

We aimed to increase trustworthiness of the study by attending to aspects of credibility, dependability, transferability, and confirmability of our findings [35]. We argue that credibility and dependability of findings were strengthened as all data were coherently and systematically analysed using inductive coding and categorization [60]. Furthermore, the parallel coding of interviews, in combination with the research team's effort to reflect on and discuss preliminary analyses ensured that categories and themes did account for all the data. We aimed to strengthen transferability by providing a rich description of context, informants, the procedures of data collection and analysis, and by providing quotations in the text representing a variety of informants [35]. Confirmability was supported by having an inquiry auditor (RS) examine the coherence between the data collected and the interpretations made [61]. In addition, we believe that the multidisciplinary research team, with occupational backgrounds in child health care, obstetrics and gynaecology, paediatrics, maternal health and maternity services research, physiotherapy, work and organizational psychology, and quality management enriched the analysis and increased the trustworthiness of the study.

One study limitation is that data could not be collected on nine eligible informants who declined participation. To what extent these non-participants were different in relation to participating informants will remain unknown. However, data collection continued until saturation was reached in order to ensure that a variety of experiences were captured. The prevalence of overweight and obesity in the sample, together with a description of lifestyle choices, could potentially have enhanced the interpretation of the results. However, a strength is that the study represents experiences from both women and men, with diverse age, lifestyles and demographic, ethnic, and socioeconomic backgrounds. Relating to level of education, our sample was well educated compared to the overall Swedish population, with 46% having a university degree compared to 24% of the population (25-64 years) [62]. Informants' experiences were also gathered through separate interviews with women and men, ensuring no influence or interference from partners.

## Conclusions

Perceptions about risks to the offspring's health appear to be the primary driving force for parental lifestyle change during pregnancy and early parenthood. However, as parents' motivation to prioritise their own health per se seems to be low during this period, future health promoting programmes need to take this into account. A more gender equal provision of health promotion to parents might increase men's involvement in lifestyle change, thus promoting a higher uptake of interventions, with health benefits for the whole family. Furthermore, parents' ranking of major lifestyle risks to the fetus may not sufficiently reflect those that constitute greatest public health concern, an area for further study.

## Competing interests

The authors declare that they have no competing interests.

## Authors' contributions

KE, IM, AI, RG, MN, and EE designed the study. KE performed the interviews, conducted the initial analysis, and drafted the manuscript in close collaboration with IM, who also coded parts of data. RS, who is bilingual in Swedish and English acted as an inquiry auditor, assisted in translation of quotes and did a linguistic review of the text. All authors contributed to the writing process before the final version was approved. All authors read and approved the final manuscript.

## Pre-publication history

The pre-publication history for this paper can be accessed here:

http://www.biomedcentral.com/1471-2458/11/936/prepub

## References

[B1] Pregnancies, Deliveries and Newborn Infants [Graviditeter, förlossningar och nyfödda barn] [In Swedish] http://www.socialstyrelsen.se/Lists/Artikelkatalog/Attachments/18267/2011-3-19.pdf

[B2] Wallby T Hjern A Child health care uptake among low-income and immigrant families in a Swedish county Acta Paediatr 2011 100 11 1495 1503 10.1111/j.1651-2227.2011.02344.x 21535134

[B3] Hildingsson I Radestad I Swedish women's satisfaction with medical and emotional aspects of antenatal care J Adv Nurs 2005 52 3 239 249 10.1111/j.1365-2648.2005.03584.x 16194177

[B4] Jungmarker EB Lindgren H Hildingsson I Playing second fiddle is okay--Swedish fathers' experiences of prenatal care J Midwifery Womens Health 2010 55 5 421 429 10.1016/j.jmwh.2010.03.007 20732663

[B5] Ortenstrand A Waldenstrom U Mothers' experiences of child health clinic services in Sweden Acta Paediatr 2005 94 9 1285 1294 16278995 10.1111/j.1651-2227.2005.tb02090.x

[B6] Hallberg AC Beckman A Hakansson A Many fathers visit the child health care centre, but few take part in parents' groups J Child Health Care 2010 14 3 296 303 10.1177/1367493510373755 20576678

[B7] Living habits. Progress report 2010. [Levnadsvanor. Lägesrapport 2010] [In Swedish] http://www.fhi.se/PageFiles/10796/A2010-13-Levnadsvanor-lagesrapport-2010.pdf

[B8] Kuh D Ben-Shlomo Y A life course approach to chronic disease epidemiology 2004 Oxford: Oxford University Press 11980781

[B9] Fowden AL Giussani DA Forhead AJ Intrauterine programming of physiological systems: causes and consequences Physiology 2006 21 29 37 10.1152/physiol.00050.2005 16443820

[B10] Gicquel C El-Osta A Le Bouc Y Epigenetic regulation and fetal programming Best Pract Res Clin Endocrinol Metab 2008 22 1 1 16 10.1016/j.beem.2007.07.009 18279777

[B11] Anzman SL Rollins BY Birch LL Parental influence on children's early eating environments and obesity risk: implications for prevention Int J Obes 2010 34 7 1116 1124 10.1038/ijo.2010.43 20195285

[B12] Fuemmeler BF Anderson CB Masse LC Parent-child relationship of directly measured physical activity Int J Behav Nutr Phys Act 2011 8 17 10.1186/1479-5868-8-17 21385455 PMC3062578

[B13] Hinkley T Crawford D Salmon J Okely AD Hesketh K Preschool children and physical activity: a review of correlates Am J Prev Med 2008 34 5 435 441 10.1016/j.amepre.2008.02.001 18407012

[B14] Savage JS Fisher JO Birch LL Parental influence on eating behavior: conception to adolescence J Law Med Ethics 2007 35 1 22 34 10.1111/j.1748-720X.2007.00111.x 17341215 PMC2531152

[B15] Thomas J Hildingsson I Who's bathing the baby? The division of domestic labour in Sweden J Family Stud 2009 15 2 139 152 10.5172/jfs.15.2.139

[B16] The Swedish Government's gender equality policy http://www.sweden.gov.se/content/1/c6/13/07/22/01f29f66.pdf

[B17] Parental benefits--insurance development and analysis [Föräldrapenning--försäkringsutveckling och analys] [In Swedish] http://www.forsakringskassan.se/irj/go/km/docs/fk_publishing/Dokument/Nyheter/110421 pressnotis/110421_nyhet.pdf

[B18] O'Brien M Fathers, parental leave policies, and infant quality of life: international perspectives and policy impact Ann Am Acad Pol Soc Sci 2009 624 190 213 10.1177/0002716209334349

[B19] Swedish Association of Obstetrics and Gynecology [Svensk Förening för Obstetrik och Gynekologi] Maternal health care, sexual and reproductive health [Mödrahälsovård, sexuell och reproduktiv hälsa] [in Swedish] Report No 59 2008 Stockholm

[B20] Hagelin E Magnusson M Sundelin C Child health care [Barnhälsovård] [In Swedish] 2007 Stockholm: Liber AB

[B21] Glanz K Rimer B Viswanath K Glanz K, Rimer B, Viswanath K Theory, research, and practice in health behaviour and health education Health behavior and health education--Theory, Research, and Practice 2008 4 San Francisco: Jossey-Bass 23 40

[B22] Marmot M Achieving health equity: from root causes to fair outcomes Lancet 2007 370 9593 1153 1163 10.1016/S0140-6736(07)61385-3 17905168

[B23] Green J Tones K Health promotion: planning and strategies 2010 London: SAGE Publications Ltd

[B24] Malterud K Qualitative research: standards, challenges, and guidelines Lancet 2001 358 9280 483 488 10.1016/S0140-6736(01)05627-6 11513933

[B25] Nystrom K Ohrling K Parenthood experiences during the child's first year: literature review J Adv Nurs 2004 46 3 319 330 10.1111/j.1365-2648.2004.02991.x 15066113

[B26] Premberg A Hellstrom AL Berg M Experiences of the first year as father Scand J Caring Sci 2008 22 1 56 63 10.1111/j.1471-6712.2007.00584.x 18269423

[B27] Deave T Johnson D The transition to parenthood: what does it mean for fathers? J Adv Nurs 2008 63 6 626 633 10.1111/j.1365-2648.2008.04748.x 18808584

[B28] Deave T Johnson D Ingram J Transition to parenthood: the needs of parents in pregnancy and early parenthood BMC Pregnancy Childbirth 2008 8 30 10.1186/1471-2393-8-30 18664251 PMC2519055

[B29] Fagerskiold A A change in life as experienced by first-time fathers Scand J Caring Sci 2008 22 1 64 71 10.1111/j.1471-6712.2007.00585.x 18269424

[B30] Thomas JE Boner AK Hildingsson I Fathering in the first few months Scand J Caring Sci 2011 25 3 499 509 10.1111/j.1471-6712.2010.00856.x 21205276

[B31] The Swedish school system: Preschool http://www.skolverket.se/polopoly_fs/1.154028!Menu/article/attachment/F%C3%B6rskola_Preschool.pdf

[B32] Dentistry in Sweden http://www.tandlakarforbundet.se/media/12957/dentistry_in_sweden.pdf?zoom_highlight=amalgam

[B33] Edvardsson K Garvare R Ivarsson A Eurenius E Mogren I Nystrom ME Sustainable practice change: Professionals' experiences with a multisectoral child health promotion programme in Sweden BMC Health Serv Res 2011 11 61 10.1186/1472-6963-11-61 21426583 PMC3077331

[B34] Eurenius E Lindkvist M Sundkvist M Ivarsson A Mogren I Maternal and paternal self-rated health and BMI in relation to lifestyle in early pregnancy--the Salut Programme in Sweden Scand J Public Health 2011 39 7 730 41 10.1177/1403494811418279 21930619

[B35] Graneheim UH Lundman B Qualitative content analysis in nursing research: concepts, procedures and measures to achieve trustworthiness Nurse Educ Today 2004 24 2 105 112 10.1016/j.nedt.2003.10.001 14769454

[B36] Open Code 3.6 http://www.phmed.umu.se/enheter/epidemiologi/forskning/open-code/

[B37] Villamor E Cnattingius S Interpregnancy weight change and risk of adverse pregnancy outcomes: a population-based study Lancet 2006 368 9542 1164 1170 10.1016/S0140-6736(06)69473-7 17011943

[B38] Davies GA Maxwell C McLeod L Gagnon R Basso M Bos H Delisle MF Farine D Hudon L Menticoglou S SOGC clinical practice guidelines: obesity in pregnancy. No. 239, February 2010 Int J Gynaecol Obstet 2010 110 2 167 173 10.1016/j.ijgo.2010.03.008 20641146

[B39] Fraser A Tilling K Macdonald-Wallis C Sattar N Brion MJ Benfield L Ness A Deanfield J Hingorani A Nelson SM Association of maternal weight gain in pregnancy with offspring obesity and metabolic and vascular traits in childhood Circulation 2010 121 23 2557 2564 10.1161/CIRCULATIONAHA.109.906081 20516377 PMC3505019

[B40] Flenady V Koopmans L Middleton P Froen JF Smith GC Gibbons K Coory M Gordon A Ellwood D McIntyre HD Major risk factors for stillbirth in high-income countries: a systematic review and meta-analysis Lancet 2011 377 9774 1331 1340 10.1016/S0140-6736(10)62233-7 21496916

[B41] Stotland NE Gilbert P Bogetz A Harper CC Abrams B Gerbert B Preventing excessive weight gain in pregnancy: how do prenatal care providers approach counseling? J Womens Health 2010 19 4 807 814 10.1089/jwh.2009.1462 PMC2867592 20078239

[B42] Edvardsson K Edvardsson D Hornsten A Raising issues about children's overweight--maternal and child health nurses' experiences J Adv Nurs 2009 65 12 2542 2551 10.1111/j.1365-2648.2009.05127.x 19824905

[B43] Campbell F Johnson M Messina J Guillaume L Goyder E Behavioural interventions for weight management in pregnancy: a systematic review of quantitative and qualitative data BMC Public Health 2011 11 1 491 10.1186/1471-2458-11-491 21696589 PMC3154865

[B44] Whitworth M Dowswell T Routine pre-pregnancy health promotion for improving pregnancy outcomes Cochrane Database Syst Rev 2009 4 CD007536 10.1002/14651858.CD007536.pub2 PMC4164828 19821424

[B45] Fleming P Godwin M Lifestyle interventions in primary care: systematic review of randomized controlled trials Can Fam Physician 2008 54 12 1706 1713 19074715 PMC2602636

[B46] Ebrahim S Taylor F Ward K Beswick A Burke M Davey Smith G Multiple risk factor interventions for primary prevention of coronary heart disease Cochrane Database Syst Rev 2011 1 CD001561 10.1002/14651858.CD001561.pub3 PMC11729147 21249647

[B47] Champion V Skinner C Glanz K, Rimer B, Viswanath K The Health Belief Model Health behavior and health education--Theory, Research, and Practice 2008 4 San Francisco: Jossey-Bass 45 65

[B48] New tools for parents--proposal for new forms of parental support [Nya verktyg för föräldrar--förslag till nya former av föräldrastöd] [In Swedish] http://www.fhi.se/PageFiles/3256/r200449nyaverktygforforaldrar.pdf

[B49] Fagerskiold A Support of fathers of infants by the child health nurse Scand J Caring Sci 2006 20 1 79 85 10.1111/j.1471-6712.2006.00383.x 16489963

[B50] Bayley J Wallace LM Choudhry K Fathers and parenting programmes: barriers and best practice Community Pract 2009 82 4 28 31 19397081

[B51] Ellberg L Hogberg U Lindh V 'We feel like one, they see us as two': new parents' discontent with postnatal care Midwifery 2010 26 4 463 468 10.1016/j.midw.2008.10.006 19084301

[B52] Finnbogadottir H Crang Svalenius E Persson EK Expectant first-time fathers' experiences of pregnancy Midwifery 2003 19 2 96 105 10.1016/S0266-6138(03)00003-2 12809629

[B53] Kreuter MW Strecher VJ Glassman B One size does not fit all: the case for tailoring print materials Ann Behav Med 1999 21 4 276 283 10.1007/BF02895958 10721433

[B54] Noar SM Benac CN Harris MS Does tailoring matter? Meta-analytic review of tailored print health behavior change interventions Psychol Bull 2007 133 4 673 693 17592961 10.1037/0033-2909.133.4.673

[B55] Oenema A Brug J Feedback strategies to raise awareness of personal dietary intake: results of a randomized controlled trial Prev Med 2003 36 4 429 439 10.1016/S0091-7435(02)00043-9 12649051

[B56] Claassen L Henneman L Janssens AC Wijdenes-Pijl M Qureshi N Walter FM Yoon PW Timmermans DR Using family history information to promote healthy lifestyles and prevent diseases; a discussion of the evidence BMC Public Health 2010 10 248 10.1186/1471-2458-10-248 20465810 PMC2875210

[B57] Miller WR Rollnick S Motivational interviewing: preparing people for change 2002 New York: Guilford Publications Inc

[B58] Rubak S Sandbaek A Lauritzen T Christensen B Motivational interviewing: a systematic review and meta-analysis Br J Gen Pract 2005 55 513 305 312 15826439 PMC1463134

[B59] Grol R Wensing M What drives change? Barriers to and incentives for achieving evidence-based practice Med J Aust 2004 180 6 Suppl S57 60 15012583 10.5694/j.1326-5377.2004.tb05948.x

[B60] Hsieh HF Shannon SE Three approaches to qualitative content analysis Qual Health Res 2005 15 9 1277 1288 10.1177/1049732305276687 16204405

[B61] Dahlgren L Emmelin M Winkvist A Qualitative methodology for international public health 2007 Umeå: Epidemiology and Public Health Sciences, Department of Public Health and Clinical Medicine, Umeå University

[B62] Educational attainment of the population 2010 [In Swedish] http://www.scb.se/Statistik/UF/UF0506/2010A01B/UF0506_2010A01B_SM_UF37SM1101.pdf

